# Polymerase Chain Reaction Assay With Urine Specimens in the Diagnosis of Acute *Chlamydia trachomatis* Infection in Women

**DOI:** 10.1155/S1064744996000531

**Published:** 1996

**Authors:** R. Pasternack, A. Mustila, P. Vuorinen, P. K. Heinonen, A. Miettinen

**Affiliations:** 1 Department of Clinical Microbiology Tampere University Hospital Box 2000 Tampere FIN-33521 Finland; 2 Department of Obstetrics and Gynecology Tampere University Hospital Tampere Finland

## Abstract

*Objective:* The purpose of this study was to evaluate the benefits achievable by Amplicor polymerase chain reaction (PCR) (F. Hoffmann-LaRoche Ltd., Basel, Switzerland) with urine specimens in addition to PACE 2 (Gen-Probe, Inc., San Diego, California) assay with cervical swab specimens in the diagnosis of *Chlamydia trachomatis* in women.

*Methods:* Cervical and urine specimens from 286 women were tested for *C. trachomatis* by PACE 2 and Amplicor PCR, respectively. All urine specimens were analyzed undiluted and diluted 1:10 to detect and eliminate possible PCR inhibition. A confirmatory PCR assay using major outer membrane protein-based primers (MOMP-PCR) was used on urine specimens that were positive by PCR from women who were negative by PACE 2 with cervical swab specimens.

*Results:* Of the endocervical specimens, 26 were positive by the PACE 2 assay. The PCR with urine was positive in 21 of these patients. When the urine specimens were analyzed diluted 1:10, 4 of the 5 PCR-negative specimens from PACE 2-positive patients turned positive by the PCR. Additionally, 4 urine specimens from PACE 2-negative women were positive by the PCR with urine, and 3 of them could be confirmed by MOMP-PCR. Altogether, 29 women were found to be positive for *C. trachomatis* by either of the two assays.

*Conclusions:* By using the PCR with urine specimens, an 11% increase in sensitivity could be achieved in addition to that obtained by PACE 2 assay with cervical swab specimens. In the present material, however, the increased sensitivity was reversed by the presence of PCR inhibitors in 14% of the female urine specimens. Amplicor PCR with urine specimens can undoubtedly be recommended for the diagnosis of chlamydial infections in women. However, constant monitoring of the PCR inhibition seems highly advisable to obtain full benefit of the sensitivity of the PCR.


Infectious Diseases in Obstetrics and Gynecology 4:276–280 (1996)
© 1997 Wiley-Liss, Inc.

### Polymerase Chain Reaction Assay With Urine Specimens in the Diagnosis of Acute *Chlamydia trachomatis* Infection in Women

R. Pasternack,[1*] A. Mustila,[1] P. Vuorinen,[1] P.K. Heinonen,[2] and A. Miettinen[1]

[1]*Department of Clinical Microbiology, Tampere University Hospital, Tampere, Finland*
[2]*Department of Obstetrics and Gynecology, Tampere University Hospital, Tampere, Finland*

ABSTRACT

*Objective:* The purpose of this study was to evaluate the benefits achievable by Amplicor polymerase chain reaction (PCR) (F. Hoffmann-LaRoche Ltd., Basel, Switzerland) with urine specimens in addition to PACE 2 (Gen-Probe, Inc., San Diego, California) assay with cervical swab specimens in the diagnosis of *Chlamydia trachomatis* in women.

*Methods:* Cervical and urine specimens from 286 women were tested for *C. trachomatis* by PACE 2 and Amplicor PCR, respectively. All urine specimens were analyzed undiluted and diluted 1:10 to detect and eliminate possible PCR inhibition. A confirmatory PCR assay using major outer membrane protein-based primers (MOMP-PCR) was used on urine specimens that were positive by PCR from women who were negative by PACE 2 with cervical swab specimens.

*Results:* Of the endocervical specimens, 26 were positive by the PACE 2 assay. The PCR with urine was positive in 21 of these patients. When the urine specimens were analyzed diluted 1:10, 4 of the 5 PCR-negative specimens from PACE 2-positive patients turned positive by the PCR. Additionally, 4 urine specimens from PACE 2-negative women were positive by the PCR with urine, and 3 of them could be confirmed by MOMP-PCR. Altogether, 29 women were found to be positive for *C. trachomatis* by either of the two assays.

*Conclusions:* By using the PCR with urine specimens, an 11% increase in sensitivity could be achieved in addition to that obtained by PACE 2 assay with cervical swab specimens. In the present material, however, the increased sensitivity was reversed by the presence of PCR inhibitors in 14% of the female urine specimens. Amplicor PCR with urine specimens can undoubtedly be recommended for the diagnosis of chlamydial infections in women. However, constant monitoring of the PCR inhibition seems highly advisable to obtain full benefit of the sensitivity of the PCR. Infect. Dis. Obstet. Gynecol. 4:276–280, 1996. © 1997 Wiley-Liss, Inc.

KEY WORDS
polymerase chain reaction; chlamydial infection; detection; female

The use of molecular biological methods in the detection of *Chlamydia trachomatis* in clinical specimens is rapidly increasing. Both an RNA hybridization assay (PACE 2, Gen-Probe, Inc., San Diego, California) and polymerase chain reaction (PCR) assay (Roche Amplicor PCR, F. Hoffmann-LaRoche, Basel, Switzerland) have been commercially available for some time. They are based, respectively, on the detection of chlamydia-specific RNA sequence directly from the clinical specimens with a nucleic acid probe or the amplification of a chlamydia-specific DNA sequence by PCR prior to the detection. Both assays have previously been compared to cell culture for the detection of endocervical chlamydial infections. The sensitivity of PACE 2 assay has been comparable to that of cell

*Correspondence to: Dr. Rafael Pasternack, Department of Clinical Microbiology, Tampere University Hospital, Box 2000, FIN-33521 Tampere, Finland.

Received 31 July 1996

Accepted 14 January 1997

culture (96.7–100%),[1,2] and the Amplicor PCR has been shown to be even more sensitive than culture.[3–5] In a recent study, we demonstrated an equal sensitivity (90% vs. 88%) between Amplicor PCR and PACE 2 performed on cervical swab specimens obtained from a high-risk population.[6]

Studies on the use of PCR with male urine specimens have demonstrated an enhanced sensitivity compared to that achieved by conventional chlamydial cell culture with urethral swab specimens.[3,4] Recently, PCR with urine specimens has been applied for the diagnosis of *C. trachomatis* infection in women.[7,8] These studies have suggested urine as a superior sample material for the detection of *C. trachomatis* also in women, and this finding has been explained by the fact that a proportion of female *C. trachomatis* infections can only be detected in the urethra. In our previous study, the presence of DNA polymerase inhibitors in 15% of female urine specimens proved to be an unexpectedly frequent threat to the performance of PCR,[8] but in other studies in different patient populations the frequency of inhibitory specimens has varied considerably, or they have not especially been searched for.

In the present study, we aimed to determine the benefit achievable by the Amplicor PCR with urine specimens in addition to PACE 2 hybridization assay with cervical specimens. Special attention was given to detecting the inhibitory urine specimens in order to evaluate the actual significance of DNA polymerase inhibitors on the sensitivity of the PCR.

### SUBJECTS AND METHODS

The study population consisted of 286 women. Their mean age was 24 years (range 15–47 years, median 22 years). Of them, 194 were enrolled for symptoms suggestive of acute chlamydial infection or with a strong suspicion of chlamydial infection because of sexual partner's proven or suspected chlamydial infection. These patients were examined and treated at the outpatient clinics of Tampere Community Health Centre and University Students' Health Care Foundation, Tampere, Finland, between May and September of 1994. In addition, 92 patients who underwent routine chlamydial sampling prior to scheduled termination of pregnancy were enrolled in the study. They were examined and treated at the Department of Obstetrics and Gynecology, Tampere University Hospital, Tampere, Finland. Patients who had received antimicrobial therapy in the preceding month were excluded from the study population. The study protocol was approved by the ethical committees of Tampere University Hospital and the City of Tampere. Informed consent was obtained from all patients.

Endocervical swab specimens were taken and transported by using the Gen-Probe Specimen Collection Kit for Cervical or Urethral Specimens. The specimens were stored at +4°C, and analyzed within 7 days from the sampling. Urine specimens for Amplicor PCR were prepared as instructed, frozen at –70°C, and analyzed within 3 weeks from the sampling.

The PACE 2 assay was performed according to the manufacturer's instructions. After RNA-DNA hybridization, the chemiluminescence signals were recorded by a LEADER-50 luminometer (Gen-Probe). The cutoff level was set relative to the mean chemiluminescence value of the three negative controls, as instructed. The probe competition assay (Gen-Probe) was used for the verification of specimens giving low chemiluminescence signals.

The Amplicor PCR was performed according to the manufacturer's instructions, and the PCR procedure was accomplished by using Perkin Elmer Thermocycler TC 9600 (Perkin-Elmer Cetus, Norwalk, Connecticut). After the amplification, the amplified nucleotide sequences were detected by an enzyme immunoassay (EIA). The absorbances were measured with a spectrophotometer (Multiscan, Labsystems Ltd., Helsinki, Finland) at a wavelength of 450 nm. Specimens giving an absorbance value of ≥0.250 were considered positive. Urine specimens repeatedly testing positive by Amplicor from women who were negative by PACE 2 in the endocervix were subjected to a confirmatory PCR assay using major outer membrane protein (MOMP)-based primers as described.[9] To detect and eliminate PCR inhibition, prepared urine specimens were further diluted 1:10 with urine dilution buffer. The diluted specimens were then analyzed according to the normal procedure.

A patient was considered to have a chlamydial infection if both PACE 2 and Amplicor PCR were positive. Additionally, if the results between PACE 2 and Amplicor PCR were discrepant specimens that could be confirmed positive either by probe

TABLE 1. Analysis of results by Gen-Probe PACE 2 RNA hybridization with endocervical specimens and Roche Amplicor PCR with urine specimens in the detection of *Chlamydia trachomatis* from 286 women

| No. of specimens | Result by Amplicor PCR with urine | Result by PACE 2 with cervical swab | Final result and conclusion |
|---|---|---|---|
| 21 | Positive | Positive | Uniformly positive |
| 3 | Positive | Negative | Positive; the positive result by PCR was confirmed by MOMP-PCR[a]; the infections were not detectable in the cervix by PACE 2 |
| 1 | Positive | Negative | Negative; the repeatedly low positive result by PCR could not be confirmed by MOMP-PCR |
| 5 | Negative | Positive | Positive; PCR inhibitors were detected in 4 urine specimens |
| 256 | Negative | Negative | Uniformly negative |

[a]Confirmatory PCR using MOMP-based primers.

competition assay or by MOMP-PCR were taken to indicate *C. trachomatis* infection. The calculations of prevalence and sensitivity are based on the total number of chlamydial infections found.

### RESULTS

The results are summarized in Table 1. Of 286 cervical specimens, 26 were positive by the PACE 2 assay. The PCR with urine was positive in 21 of these patients. Additionally, 4 urine specimens from PACE 2-negative women were positive by the PCR. Of the 4, 3 could be confirmed as true positive by MOMP-PCR, whereas one urine specimen which was repeatedly weakly positive by PCR (absorbance values of 0.35–0.5) remained negative by MOMP-PCR. Two of the PCR-positive women who were negative by PACE 2 had not received antibiotics at their initial visit and were subjected to second sampling about 2 weeks later. Both of them were still positive by PCR with urine and, in addition, both had also turned positive by PACE 2 with endocervical swabs.

When the prepared urine specimens were diluted 1:10 for the PCR, 4 of the 5 PCR-negative specimens from PACE 2-positive patients turned positive by the PCR, indicating the presence of PCR inhibitors. In contrast, 3 urine specimens from PACE 2-positive patients that were originally positive by the PCR turned negative when diluted, indicating a substantial loss of sensitivity by the additional dilution procedure.

When the total number of 29 chlamydial infections detected was regarded as the basis of calculation, the use of PCR with female urine specimens yielded a sensitivity of 82.8%. The sensitivity of PACE 2 assay with endocervical specimens was 89.7%. By using both diluted and undiluted urine specimens to eliminate PCR inhibition, the sensitivity of PCR with urine could be enhanced to 96.6%.

Amplicor PCR detected *C. trachomatis* in the urine specimens of 4 women that were negative by PACE 2 with cervical specimens. Three of these 4 were confirmed positive by MOMP-PCR. The confirmed chlamydial infections that could only be found in urine thus represent 10% of the total number of infections found in the present study.

### DISCUSSION

Earlier studies have shown that urine is inappropriate for chlamydial isolation.[10] Various antigen detection tests, on the other hand, have been successfully applied for the detection of *C. trachomatis* infection in male urine with a sensitivity approximating that achieved by culturing urethral swab specimens.[11,12] The fact that similar testing of female urine did not approximate results achieved by culturing cervical swabs was explained by assuming that not all patients with endocervical infection had infected urethras.[12,13] In clinical practice, urethral sampling in females is frequently overlooked.

PCR on male urine specimens has been shown to be a highly sensitive and specific non-invasive technique for the diagnosis of *C. trachomatis*, providing an opportunity for the identification of both asymptomatic and symptomatic infected patients.[3,4] According to recent studies, the amplification power of PCR appears to make possible the same approach in females as well.[7,8]

The presence of DNA polymerase inhibitors was first described in endocervical specimens, where the ensuing percentage of false negative results varied between 4% and 10% in different reports, depending on the prevalence of chlamydial infection in the population studied.[4,5] PCR inhibitors were also found in male urine although they were first thought to occur less frequently than in endocervical specimens, i.e., in 0–3% of true positive specimens.[3,4] Wiesenfeld et al.[14] reported the presence of PCR inhibitors in almost 10% of positive male urine specimens in a high-risk population with 17% incidence of chlamydial infection. In a recent study performed in a low-risk female population, PCR inhibitors were encountered in 15% of the resolved positive female urine specimens, which is in complete agreement with the 14% prevalence of PCR inhibition among the true positive specimens in the current high-risk female population.[8]

Clinical specimens may contain inhibitors of the DNA polymerase with varying frequency. The exact nature of the PCR inhibitors is unknown, and their presence cannot be predicted from the characteristics of the patient or the specimen. Several methods including dilution and repeated freezing and thawing have been used to eliminate them but so far, no straightforward guidelines exist for their elimination. The present study clearly showed that the additional dilution of the urine specimens can be harmful for the sensitivity of the detection and cannot be recommended for routine testing. The testing of all specimens both undiluted and diluted, on the other hand, will double the reagent and labor costs and is not tempting for routine laboratories. An internal control procedure integrated as part of the PCR assay will probably prove to be an indispensable feature in the newly launched automated PCR applications.

In conclusion, PCR with urine specimens offers a tempting non-invasive technique for the diagnosis of *C. trachomatis* infection in females. In this study, it was used as an extension of the normal routine in our laboratory employing PACE 2 assay on cervical swab specimens. Although the current patient material consisted of two separate groups, the prevalence of chlamydial infection in these groups was equal and no difference in the best performance between these groups could be demonstrated. The majority of infections found by PACE 2 could also be found in urine by PCR. Moreover, an 11% increase in the yield of positive results could be achieved by the analysis of urine by PCR, probably representing patients with chlamydial infection restricted to the urethra. By using the current modification of Amplicor PCR, however, the sensitivity of detection was seriously compromised by the frequent presence of PCR inhibitors in urine. In fact, the resulting false negative results outnumbered the positive cases that were only detectable by the PCR assay with urine specimens. PCR with urine specimens can undoubtedly be recommended for the diagnosis of chlamydial infections in women. However, constant monitoring of the PCR inhibition seems to be a prerequisite for obtaining full benefit of the sensitivity of the PCR.

### ACKNOWLEDGMENTS

We gratefully acknowledge the effort of K. Becker, Roche Diagnostic Systems, PCR Business Development Europe, for performing the confirmatory MOMP-PCR assays. The study was supported by a grant from the Medical Research Fund of Tampere University Hospital, Tampere, Finland.

### REFERENCES

1. Warren R, Dwyer B, Plackett M, Pettit K, Rizvi N, Baker A-M: Comparative evaluation of detection assays for *Chlamydia trachomatis*. J Clin Microbiol 31:1663–1664, 1993.
2. Stary A, Teodorowicz L, Hörting-Müller I, Nerad S, Storch M: Evaluation of the Gen-Probe PACE 2 and the Microtrak enzyme immunoassay for diagnosis of *Chlamydia trachomatis* in urogenital samples. Sex Transm Dis 21:26–30, 1994.
3. Jaschek G, Gaydos CA, Welsh LA, Quinn TC: Direct detection of *Chlamydia trachomatis* in urine specimens from symptomatic and asymptomatic men by using a rapid polymerase chain reaction assay. J Clin Microbiol 31:1209–1212, 1993.
4. Bauwens JE, Clark AM, Loeffelholz MJ, Herman SA, Stamm WE: Diagnosis of *Chlamydia trachomatis* urethritis in men by polymerase chain reaction assay of first-catch urine. J Clin Microbiol 31:3013–3016, 1993.
5. Bass CA, Jungkind DL, Silverman NS, Bond JM: Clinical evaluation of a new polymerase chain reaction assay for detection of *Chlamydia trachomatis* in endocervical specimens. J Clin Microbiol 31:2648–2653, 1993.
6. Miettinen A, Vuorinen P, Varis T, Hällström O: Comparison of enzyme immunoassay antigen detection, nucleic acid hybridization, and polymerase chain reaction assay in *Chlamydia trachomatis* infection. Eur J Clin Microbiol Infect Dis 14:546–549, 1995.
7. Skulnick M, Chua R, Simor AE, et al.: Use of the polymerase chain reaction for the detection of *Chlamydia trachomatis* from endocervical and urine specimens in an asymptomatic low-prevalence population of women. Diagn Microbiol Infect Dis 20:195–201, 1994.
8. Pasternack R, Vuorinen P, Kuukankorpi A, Pitkäjärvi T,

Miettinen A: Detection of *Chlamydia trachomatis* infection in women by Amplicor PCR: Comparison of diagnostic performance with urine and cervical specimens. J Clin Microbiol 34:995–998, 1996.

9. Dutilh B, Bebear C, Rodriguez P, Vekris A, Bonnet J, Garret M: Specific amplification of a DNA sequence common to all *Chlamydia trachomatis* serovars using a polymerase chain reaction. Res Microbiol 140:7–16, 1989.
10. Smith TF, Weed LA: Comparison of urethral swabs, urine, and urinary sediment for the isolation of *Chlamydia.* J Clin Microbiol 2:134–135, 1975.
11. Moncada J, Schachter J, Shafer M-A, et al.: Detection of *Chlamydia trachomatis* in first catch urine samples from symptomatic and asymptomatic males. Sex Transm Dis 21:8–12, 1994.
12. Chernesky M, Castriciano S, Sellors J, et al.: Detection of *Chlamydia trachomatis* antigens in urine as an alternative to swabs and cultures. J Infect Dis 161:124–126, 1990.
13. Bradley MG, Hobson B, Lee N, Tait IA, Rees E. Chlamydial infections of the urethra in women. Genitourin Med 61:371–375, 1985.
14. Wiesenfeld HC, Uhrin M, Dixon BW, Sweet RL: Diagnosis of male *Chlamydia trachomatis* urethritis by polymerase chain reaction. Sex Transm Dis 21:268–271, 1994.
